# Sustainable Water- and Oil-Repellent Coating for Disposable Meal Boxes Based on Highly Deacetylated Chitosan

**DOI:** 10.3390/ma18122741

**Published:** 2025-06-11

**Authors:** Zhiwei Shen, Yihan Yang, Shufeng Hu, Weiqing Kong

**Affiliations:** State Key Laboratory of Advanced Fiber Materials, College of Materials Science and Engineering, Donghua University, Shanghai 201620, China; szw709133136@163.com (Z.S.); 221100317@mail.dhu.edu.cn (Y.Y.); shufengbeyond@yeah.net (S.H.)

**Keywords:** chitosan, degree of deacetylation, fluorine free, water and oil repellent

## Abstract

To mitigate the serious environmental impact caused by the persistent accumulation of plastics, replacing conventional plastics with paper-based alternatives has emerged as a promising trend. In response to the environmental and health concerns associated with petrochemical-based plastic meal boxes and fluorinated water- and oil-repellent agents, this study proposes a sustainable, fluorine-free coating technology based on chitosan to enhance the water and oil resistance of molded-paper pulp meal boxes. By adjusting the degree of deacetylation and the solution concentration of chitosan, coated meal boxes were fabricated via a spraying method. The results demonstrate that coatings prepared with highly deacetylated (>95%) and concentrated (4% *w*/*v*) chitosan significantly improve barrier properties, achieving a water contact angle of 114.9° ± 3°, the highest oil-resistance rating (12/12) according to TAPPI standards, and stable resistance to 95 °C hot oil for up to 30 min without leakage. In addition, the coated samples exhibit enhanced mechanical strength (21.26 MPa) and excellent biodegradability. This work provides a cost-efficient and eco-friendly disposable food packaging solution, facilitating the sustainable substitution of petrochemical-based plastics.

## 1. Introduction

The annual global production of plastics has exceeded 400 million tons, with the food packaging sector accounting for approximately 36% of this total. However, most conventional plastic materials are non-biodegradable, resulting in the accumulation of persistent plastic waste and the widespread emergence of microplastic pollution, which has even been detected in polar glaciers. To mitigate this growing environmental crisis, policy measures have been enacted worldwide. In 2023, the European Union officially adopted the PFAS Restriction Proposal, mandating a comprehensive ban on fluorinated food contact materials by 2025. In parallel, China’s “14th Five-Year Action Plan for Plastic Pollution Control” also calls for accelerating the adoption of paper-based alternatives. Against this backdrop, the development of molded-pulp packaging materials with excellent water and oil resistance, full biodegradability, and environmental safety has become an urgent necessity in the green packaging industry.

Cellulose is the most abundant renewable resource in nature, possessing advantages such as non-toxicity, degradability, and recyclability, making it an ideal alternative substrate for plastics [1,2]. However, pulp molding materials based on cellulose exhibit amphiphilicity due to their rich hydroxyl groups and porous structure, resulting in insufficient natural water and oil repellency, which limits their application in fields with high barrier requirements such as food packaging [3,4,5].

To address this limitation, traditional methods such as plastic coatings or synthetic fluorinated agents have been widely used. Although effective in enhancing barrier properties, plastic coatings often incorporate non-degradable petroleum-based materials, aggravating microplastic contamination [6]. Meanwhile, fluorine-containing water and oil repellents function by lowering surface energy but introduce long-term environmental risks. Perfluorinated compounds such as PFOS and PFOA are known for their extreme environmental persistence, bioaccumulation potential, and toxicity, prompting their prohibition in food contact applications by multiple regulatory bodies [7,8,9]. Therefore, there is an urgent need for fluorine-free, biodegradable, and renewable polymer-based barrier solutions to achieve both functional performance and environmental sustainability.

Among bio-based materials, starch, nanocellulose, and chitosan have emerged as promising candidates for green barrier coatings [10,11,12]. Starch, owing to its low cost and good film-forming ability, is commonly applied in surface sizing and oil-resistance treatments [13,14]. However, its standalone oil repellency is limited, and it typically requires formulation with other polymers or functional additives to enhance performance [15,16]. Dong Kaihui [16] prepared a compound oil-proofing agent by mixing cationic starch (CS) and carboxymethyl cellulose (CMC) in a mass ratio of 1:1 and combined it with an environmentally friendly oil-proofing agent. The resulting agent was then applied to the base paper to improve its oil-proofing performance. The results showed that as the proportion of the compound oil-proofing agent increased, the oil-proofing performance also improved. When the ratio of the compound oil-proofing agent to the environmentally friendly oil-proofing agent was 4:1, the oil-proofing grade reached a maximum of 9/12. Nanocellulose exhibits good biocompatibility and barrier potential, but its film-forming compactness is insufficient, and its oil-proofing ability is inferior to that of chitosan [17]. In contrast, chitosan, derived from the deacetylation of chitin, possesses strong film-forming properties, excellent antibacterial properties, and superior oil-barrier capabilities, demonstrating unique advantages in the field of food packaging [18,19,20]. The amino and hydroxyl groups in its molecule can form a dense hydrogen bond network, effectively filling the pores of the cellulose substrate and enhancing the overall barrier performance of the material [21,22].

However, existing research has mostly focused on the performance evaluation of chitosan at a single concentration or degree of deacetylation, with a lack of systematic exploration into the synergistic regulation mechanism between different degrees of deacetylation and concentrations. Furthermore, the stability, mechanical strength, and degradation behavior of chitosan coatings in high-temperature oil–water environments and in practical application scenarios have not been fully verified. For example, Kjellgren et al. [23] only studied chitosan coatings with a concentration of 1 wt% and a degree of deacetylation of 85%. The results showed that only when the coating weight of the chitosan solution reached 5 g m^−2^ could it successfully act as a barrier to oil and grease. Ham-Pichavant et al. [22] enhanced the oil resistance of sodium alginate through composite reinforcement. However, due to the high viscosity of the system, it is difficult to carry out industrial sizing, and there is an urgent need to optimize the formula to balance performance and process adaptability.

To address these limitations, the present study investigates the effects of chitosan coatings with varying degrees of deacetylation (80–95% and >95%) and concentrations (2%, 3%, 4% *w*/*v*) on the functional performance of molded-pulp meal boxes. A spray-coating method is employed to achieve uniform deposition, and the resulting samples are systematically evaluated in terms of water and oil repellency, thermal resistance, mechanical strength, and biodegradability. The roles of chitosan concentration and deacetylation in forming surface films, filling matrix pores, and establishing hydrogen-bonded interfacial networks are elucidated through water/oil contact angle measurements, oil-resistance grading, and heat-endurance testing. Furthermore, ATR-FTIR spectroscopy, scanning electron microscopy (SEM), and thermogravimetric analysis (TGA) are used to explore the molecular-level interactions between chitosan and the cellulose matrix. This work not only deepens the mechanistic understanding of chitosan-based barrier coatings but also provides practical guidance for developing scalable, fluorine-free, and environmentally friendly food packaging materials, supporting the global transition from plastic to paper-based alternatives.

## 2. Materials and Methods

### 2.1. Materials

Chitosan (80–95% deacetylated) was purchased from Sinopharm. Chitosan (>95% deacetylated, viscosity = 100–200 mPa s) was purchased from ALADDIN, Shanghai, China. Glacial acetic acid (≥99.5%) was purchased from Sinopharm, Beijing, China. All the water used in the experiment was deionized water. Meal box specifications: Supplier: Green Cycle New Materials (Yunnan) Co., Ltd., Lincang, China. Meal box specifications: Basis weight (454.1658 g/m^2^), thickness (0.64 mm), and density (0.7096 g/cm^3^). All meal boxes were pre-dried at 60 °C for 2 h before chitosan coating to minimize residual moisture interference.

### 2.2. Preparation of Chitosan Solutions with Varying Degrees of Deacetylation

Chitosan solutions with different degrees of deacetylation (DD) and concentrations were prepared as follows. For the high-DD chitosan (DD > 95%), 4.0 g of chitosan was gradually added to a 2% (*v*/*v*) acetic acid aqueous solution under continuous stirring, and the total volume was adjusted to 100 mL with deionized water to obtain a 4% (*w*/*v*) chitosan solution. The mixture was stirred at room temperature for 12 h to ensure complete dissolution and then left undisturbed for 24 h to eliminate air bubbles and obtain a homogeneous solution.

Chitosan solutions with lower concentrations (2% and 3% *w*/*v*) were prepared using the same protocol by adjusting the amount of chitosan accordingly. For medium-DD chitosan (DD = 80–95%), the same procedure was employed to prepare 2%, 3%, and 4% (*w*/*v*) solutions in 2% acetic acid, ensuring consistent preparation conditions across all samples.

### 2.3. Preparation of Chitosan-Coated Molded-Pulp Lunch Boxes

Chitosan solutions with varying degrees of deacetylation and concentrations were applied to the surface of molded-pulp lunch boxes using a spray-coating method. Specifically, each chitosan solution was uniformly sprayed onto the sample surface using an air compressor (Level 2 1100, Taizhou AOTUSI Industrial & Trade Co., Ltd., Taizhou, Zhejiang, China) equipped with a spray gun (W-101, Iwasaki, Japan), followed by drying in a constant-temperature oven at 80 °C for 3 h to ensure complete film formation and adhesion. The air compressor pressure was 0.7 MPa and the nozzle diameter was 1.5 mm.

All chitosan solutions—regardless of deacetylation degree (80–95% and >95%) and concentration (2%, 3%, and 4% *w*/*v*)—were processed under identical conditions to ensure comparability. Uncoated lunch boxes were used as the blank control group.

### 2.4. Material Basis Weight, Coating Load, and Material Thickness

The basis weight (unit area mass, g m^−2^) of uncoated and chitosan-coated samples was measured according to ASTM D646. Specifically, uncoated samples (Un), 80–95% deacetylated chitosan-coated samples (80C), and >95% deacetylated chitosan-coated samples (95C) were each cut into squares with dimensions of 200 ± 15 mm × 200 ± 15 mm. The mass of each sample was recorded both before and after coating using an analytical balance. The average basis weight of each sample was calculated using Equation (1), which represents the ratio of the sample mass to its area.

The coating load was determined by calculating the difference in weight between the coated and uncoated samples, as shown in Equation (2). The thickness of each sample was measured at three different locations using a digital thickness gauge. The measurements were recorded in micrometers (μm), and the final reported thickness value was the average of the three individual readings.

(1) $$\mathrm{Basis}\;\mathrm{weight}=\frac{\mathrm{weight}(g)}{\mathrm{area}(m^{2})}$$

(2) Coating load=basis weight∗(mass of coated−uncoated)

### 2.5. Water and Oil Contact Angle Test (CA)

The contact angles (CAs) of water and edible oil were measured on Un, 80C, and 95C samples using an OCA40 Micro (Dataphysics, Filderstadt, Germany)—a fully automatic, video instrument for measuring micro contact angles. A 3 μL droplet of water and a 5 μL droplet of edible oil were placed at three random positions on the surface of each sample. The contact angles were recorded at two different time intervals: 30 s and 300 s, after droplet deposition. Photographs were taken and contact angle data were recorded at each time point.

### 2.6. Grease Resistance

The grease resistance of the Un, 80C, and 95C samples was evaluated according to the TAPPI T559 pm-96 standard protocol. A mixture of castor oil, toluene, and n-heptane was prepared in varying proportions to create 12 different solution grades, representing a range of grease resistance from poor to excellent. Solutions were assigned grades 1 through 12, with higher numbers indicating better resistance.

To perform the test, a specific grade of solution was dropped onto the surface of the sample from a height of 13 mm and left for 15 s. After this time, the solution was wiped off. If no oil stains remained on the surface, the sample passed the oil-resistance test for that grade, and testing continued with a higher-grade solution. The process was repeated until the sample failed to pass a given grade. The grease-resistance grade of the sample was assigned based on the highest solution grade passed. The average of three experimental trials for each sample was calculated to determine the final grease-resistance rating. Table 1 presents the preparation methods for the 12 different solution grades.

### 2.7. Hot Water and Oil Resistance Test

In accordance with the GB/T 36787-2018 standard [24], the hot-water and oil resistance of the Un, 80C, and 95C samples were evaluated. The test was performed by placing the sample on a dry glass plate or a flat surface lined with filter paper, followed by the addition of water heated to 95 ± 5 °C. The sample was then allowed to sit for 30 min. After this period, the sample was inspected for any deformation or leakage at the bottom. Three replicate tests were conducted for each sample. If none of the three samples showed deformation or leakage, the sample was considered to have passed the hot water-resistance test without deformation or leakage. It is important to note that condensation of water vapor at the bottom of the sample due to temperature differences between the interior and exterior is not considered a leak.

For the heat-resistant oil test, the sample was placed on a dry glass plate or flat surface lined with filter paper. Edible oil, heated to 95 ± 5 °C, was added to the sample, and it was allowed to stand for 30 min. During this time, the sample was observed for any signs of deformation or leakage, and the filter paper was checked for oil stains. Each sample underwent three replicates of this test. If none of the three samples exhibited deformation or leakage, and no oil stains were present on the filter paper, the sample was considered to have passed the heat-resistant oil test.

### 2.8. Chitosan Viscosity Test

The viscosity of chitosan solutions with varying degrees of deacetylation and concentrations was measured using an MCR302 rheometer (Anton Paar MCR302, Graz, Austria). The viscosity measurements were conducted at room temperature by varying the shear rate from 0.01 to 1000 s^−1^. This analysis aimed to investigate the influence of chitosan viscosity on key performance metrics, including water and oil repellency, mechanical strength, and coating uniformity.

### 2.9. Fourier Transform Infrared Spectroscopy (ATR-FTIR) Analysis

The chemical interactions between the chitosan coatings and the cellulose substrate were characterized using a Nicolet iS50 Fourier Transform Infrared Spectrometer equipped with an Attenuated Total Reflection (ATR) accessory (Thermo Scientific, Nicolet iS50, Waltham, MA, USA). The infrared spectra of Un, 80C, and 95C samples were recorded. Each spectrum was collected by averaging 32 scans within the spectral range of 4000–400 cm^−1^ to ensure a sufficient signal-to-noise ratio and spectral resolution.

### 2.10. Scanning Electron Microscope (SEM)

The surface and cross-sectional morphologies of Un, 80C, and 95C samples were examined using scanning electron microscopy (SEM, Hitachi, SU8010, Tokyo, Japan). Prior to observation, the samples were sputter-coated with a thin layer of gold–palladium to enhance conductivity. SEM imaging was conducted at an accelerating voltage of 3 kV and a working distance of 8 mm to ensure the high-resolution visualization of coating uniformity, surface compactness, and interfacial adhesion.

### 2.11. Mechanical Properties

The mechanical properties of Un, 80C, and 95C samples were evaluated using a CTM2050 microcomputer-controlled electronic universal testing machine (Xieqiang Instruments Manufacturing Co., Ltd., Shanghai, China). The parameters measured included tensile strength, elastic modulus, and fracture strength.

Samples were cut into rectangular strips of 40 mm × 15 mm and mounted onto the machine grips with a gauge length of 20 mm. The specimens were stretched at a constant crosshead speed of 20 mm min^−1^ until failure. Each sample group was tested three times, and the average values were calculated and reported to ensure statistical reliability.

### 2.12. Biodegradation Performance

The biodegradability of the Un, 80C, and 95C samples was evaluated using a soil burial test. Samples were buried in natural soil at a depth of approximately 5 cm under controlled ambient conditions to simulate real-world composting environments. The buried samples were retrieved at predetermined intervals of 15, 60, and 120 days. Upon excavation, the samples were gently cleaned, photographed, and visually examined to assess morphological changes and surface degradation as indicators of biodegradation.

### 2.13. Thermal Gravimetry (TGA)

The TG 209 F1 thermogravimetric analyzer was used to conduct thermal gravimetric analysis (TGA) on samples of 80C and 95C. Samples weighing 1–5 mg were heated at a constant rate of 10 °C/min from 30 °C to 600 °C in an N_2_ atmosphere to obtain the thermal gravimetric analysis curves. The first-order derivatives of these curves were calculated to obtain the derivative thermal gravimetric analysis curves (DTG).

## 3. Results and Discussion

**Figure 1:**
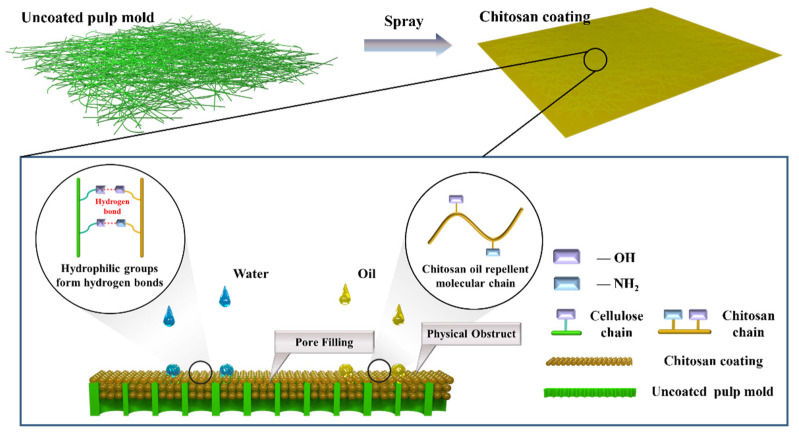
Mechanism of enhanced water and oil repellency of pulp meal boxes via chitosan coating.

The molecular structures of cellulose and chitosan are illustrated in Figure 1, and the proposed mechanism by which chitosan enhances the water and oil repellency of the coated samples is shown schematically in Figure 1.

### 3.1. Material Basis Weight, Coating Load, and Material Thickness

The basis weight, coating load, and material thickness are fundamental physical properties that influence the mechanical strength, barrier performance, and practical applicability of the materials. The measured values for the uncoated and chitosan-coated samples are summarized in Table 2. The thickness of the uncoated sample was 670 μm. After coating with chitosan solutions, the thickness increased to a range of 682–687 μm, indicating that the thickness of the single-layer chitosan coating was approximately 15 μm. Notably, samples coated with 80–95% deacetylated chitosan exhibited slightly greater thickness compared to those coated with >95% deacetylated chitosan. This difference is attributed to the lower viscosity of the 80–95% chitosan solution, which resulted in a higher spray volume during the coating process.

### 3.2. Water and Oil Contact Angle

**Figure 2:**
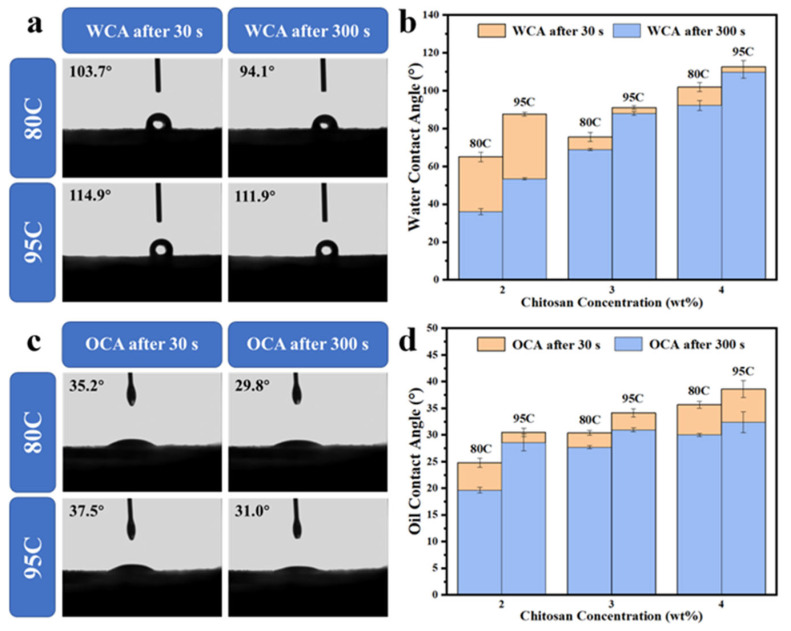
(**a**) Water contact angles of chitosan-coated samples with different degrees of deacetylation measured at 30 s and 300 s after droplet deposition. (**b**) Comparison of water contact angles for chitosan coatings with different concentrations (2%, 3%, 4% *w*/*v*) and deacetylation degrees at 30 s and 300 s. (**c**) Oil contact angles of chitosan-coated samples with different degrees of deacetylation measured at 30 s and 300 s after droplet deposition. (**d**) Comparison of oil contact angles for chitosan coatings with different concentrations and deacetylation degrees at 30 s and 300 s.

The water contact angles of chitosan-coated samples with varying degrees of deacetylation were measured using a contact angle goniometer to evaluate their water repellency, as shown in Figure 2a. Two sets of contact angle data were recorded: one at 30 s and the other at 300 s after water droplet deposition. Additionally, the effect of both deacetylation degree and chitosan solution concentration on the water contact angle was compared at the two time points, as illustrated in Figure 2b.

The results showed that the uncoated samples exhibited no water repellency and with water droplets being fully absorbed within 30 s, resulting in a contact angle of 0°. This poor performance is attributed to the polar hydroxyl groups and porous structure inherent in pulp molding [25]. For the samples coated with chitosan of 80–95% deacetylation (80C), the water contact angle was 103.7° at 30 s and decreased to 94.1° at 300 s, indicating some loss in hydrophobic performance over time. In contrast, samples coated with highly deacetylated chitosan (>95%, 95C) showed superior water resistance, maintaining a contact angle of 114.9° at 30 s and 111.9° at 300 s. This result demonstrates that a higher degree of deacetylation improves the long-term water repellency of the coating, likely due to enhanced film-forming ability and better pore-filling behavior on the cellulose surface. Moreover, increasing the chitosan concentration from 2% to 3% and 4% (*w*/*v*) led to a progressive rise in contact angle values. Higher-concentration coatings more effectively sealed surface pores and improved surface uniformity, thereby enhancing the overall barrier performance. These findings indicate that within a certain range, both higher deacetylation degree and increased concentration contribute positively to the water repellency of the chitosan-coated molded-pulp materials.

As shown in Figure 2c, the oil contact angles of chitosan-coated samples with different degrees of deacetylation were measured. The effect of both deacetylation degree and coating concentration on oil contact angle was further analyzed at 30 and 300 s, as presented in Figure 2d.

The uncoated sample (Un) showed no oil repellency, with the oil contact angle decreasing to 0° within 30 s. Upon chitosan coating, a notable improvement in oil resistance was observed. For samples coated with chitosan of 80–95% deacetylation (80C), the oil contact angle was 35.2° at 30 s and decreased to 29.8° at 300 s. In comparison, samples coated with highly deacetylated chitosan (95C) exhibited slightly better performance, with contact angles of 37.5° and 31.0° at 30 and 300 s, respectively.

Despite this improvement, the absolute oil contact angle values remained relatively low and did not fully reflect the actual oil and grease barrier performance observed in practical applications. This apparent discrepancy can be attributed to the inherent surface tension mismatch between chitosan films and non-polar oil droplets. Specifically, chitosan has a relatively high surface energy, which promotes the lateral spreading of oil droplets on the surface, resulting in a lower measured contact angle even in the presence of effective oil repellency.

Analysis of the data indicates that increasing the degree of deacetylation slightly improves oil repellency, although the enhancement is limited. By contrast, increasing the chitosan concentration proves to be a more effective approach for enhancing oil contact angles, likely due to thicker and more continuous coatings that reduce oil permeation. Nevertheless, due to the fundamental surface properties of chitosan, the measured oil contact angles still fall short of ideal values, underscoring the limitations of contact angle measurements as the sole indicator of oil barrier performance.

### 3.3. Oil Resistance Level Testing

**Figure 3:**
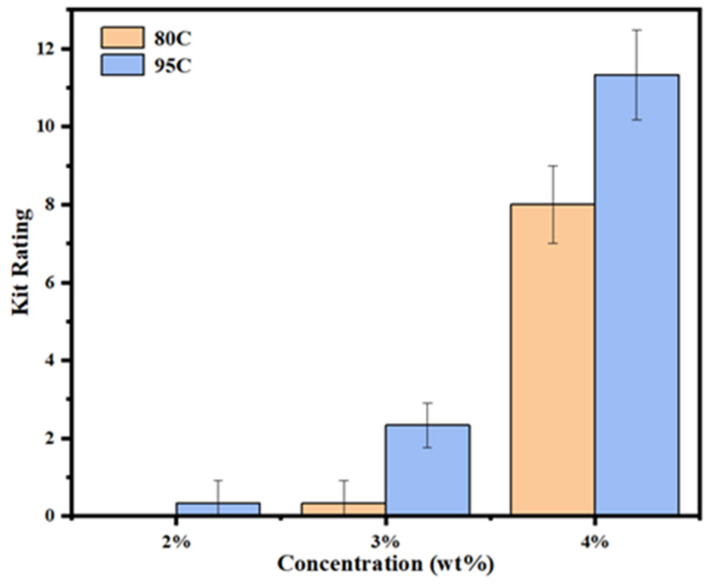
Kit rating test of chitosan-coated meal boxes with different degrees of deacetylation and concentrations.

The oil resistance of chitosan-coated samples with different degrees of deacetylation and concentrations was evaluated according to the TAPPI T559 pm-96 standard method. Twelve test solutions with varying surface tensions were used, and the oil-resistance level was recorded on a scale from 0 to 12, with higher values indicating better oil repellency. The results are presented in Figure 3.

The uncoated sample failed to pass the first-level test (0/12) and was therefore excluded from the figure. As shown in the data, chitosan coating significantly improved the oil resistance of the samples. For chitosan with a deacetylation degree > 95%, the 2% (*w*/*v*) coated sample successfully surpassed the baseline level, achieving an oil-resistance level of 1/12. The 3% coated sample reached 3/12, and the 4% coated sample exhibited the highest performance with an oil-resistance level of 12/12.

In contrast, samples coated with 80–95% deacetylated chitosan failed the first-level test at a 2% concentration. However, the 3% coated sample achieved a resistance level of 1/12, and the 4% coated sample demonstrated a marked improvement, reaching 8/12. These results suggest that increasing both the deacetylation degree and concentration of chitosan can effectively enhance the oil barrier performance of coated samples.

Chitosan enhances the oil resistance of the substrate primarily through its inherent polarity and its ability to fill the porous structure of the base material. At low concentrations (2%), the chitosan coating is insufficient to adequately penetrate and seal the pores on the substrate surface, resulting in only minimal improvement in oil repellency. As the coating concentration increases, especially at 4%, the chitosan effectively fills the inter-fiber voids within the substrate, leading to a substantial enhancement in oil-resistance level.

The degree of deacetylation of chitosan significantly influences the oil repellency of the coated samples, in a manner similar to the effect of concentration. Chitosan with a deacetylation degree between 80 and 95% is categorized as high deacetylated chitosan, while chitosan with a deacetylation degree greater than 95% is classified as ultra-high deacetylated chitosan. Increasing the deacetylation degree enhances the solubility and film-forming properties of chitosan in acetic acid solution, which improves its ability to fill the pores of the substrate, ultimately enhancing its oil and fat barrier properties. Furthermore, higher deacetylation degrees result in a higher concentration of amino groups in the chitosan molecules. Under acidic conditions, these amino groups become protonated and positively charged, facilitating the adsorption of oils and fats [22,26]. Although chitosan inherently has a lower oil contact angle due to its high surface tension, practical applications show that chitosan-coated samples exhibit excellent oil and fat resistance, achieving the highest oil-resistance level of 12/12 in the oil-resistance level test.

### 3.4. Hot Water and Hot Oil Resistance Performance

**Figure 4:**
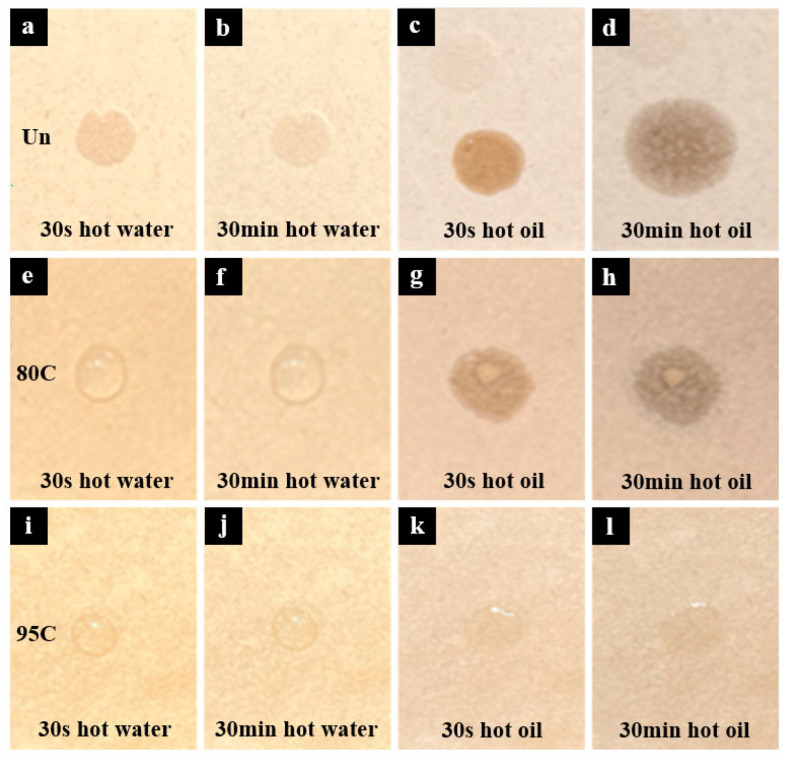
Photos of hot water dripping into the original meal box (Un), 80–95% deacetylated chitosan-coated meal box (80C), and >95% deacetylated chitosan-coated meal box (95C) after 30 s (**a**,**e**,**i**) and 30 min (**b**,**f**,**j**); Photos of hot oil dripping into the original meal box (Un), 80–95% deacetylated chitosan-coated meal box (80C), and >95% deacetylated chitosan-coated meal box (95C) after 30 s (**c**,**g**,**k**) and 30 min (**d**,**h**,**l**).

To evaluate the resistance of different meal boxes to hot water and hot oil, water and oil at 95 ± 5 °C were, respectively, dropped onto three types of samples: Un, 95C, and 80C. Photographs were taken after 30 s and 30 min of contact, as shown in Figure 4. As shown in Figure 4a–d, the uncoated sample was quickly penetrated by both hot water and hot oil within 30 s, indicating a complete lack of water and oil repellency. In contrast, Figure 4e–h shows that the chitosan-coated sample with a deacetylation degree of 80–95% exhibited poor resistance to hot oil due to insufficient pore filling, resulting in oil infiltration. However, it still maintained good resistance to hot water, showing no significant penetration even after 30 min. The sample coated with chitosan of >95% deacetylation degree demonstrated superior pore-filling capability and formed a more uniform film, providing effective barrier properties against both hot water and hot oil for at least 30 min. Moreover, as shown in Figure 4i–l, the contact angle of oil droplets was significantly smaller than that of water droplets. This phenomenon is attributed to the lower surface tension of oil compared to water, leading to a greater tendency of oil to spread on the substrate surface.

**Figure 5:**
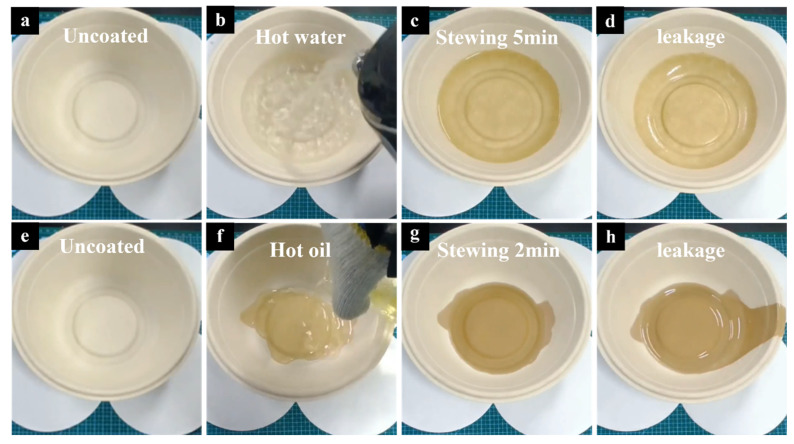
(**a**–**d**) Hot water-resistance test of the uncoated meal box. (**e**–**h**) hot oil-resistance test of the uncoated meal box.

Figure 5 shows the results of the hot water- and hot oil-resistance tests for the uncoated sample. It can be observed that after the application of hot water and hot oil (95 ± 5 °C), the uncoated sample exhibited leakage within a short period of time—approximately 2 min for hot water and 5 min for hot oil—indicating failure in both the hot water- and hot oil-resistance tests.

**Figure 6:**
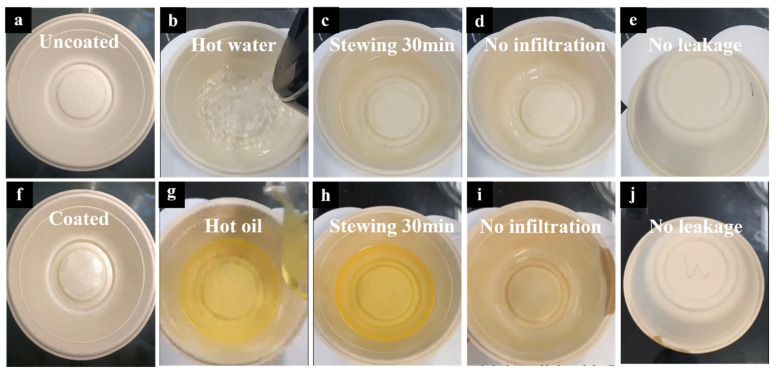
(**a**) Uncoated meal box. (**f**) Chitosan-coated meal box. (**b**–**e**) Hot water-resistance test of chitosan-coated meal boxes. (**g**–**j**) Hot oil-resistant test of chitosan-coated meal boxes.

Figure 6 presents the hot water- and hot oil-resistance performance of the chitosan-coated sample. Over a 30 min observation period, the coated sample maintained excellent barrier properties, showing no signs of leakage from the interior or the bottom. In addition, as shown in the Figure 6i, the edge of the meal box, which was not coated with chitosan, exhibited noticeable oil leakage, in sharp contrast to the internal surface where chitosan coating effectively prevented oil penetration. These results confirm that the chitosan coating significantly enhances the thermal liquid resistance of the substrate, enabling it to pass both hot water- and hot oil-resistance tests.

### 3.5. Chitosan Viscosity Testing

**Figure 7:**
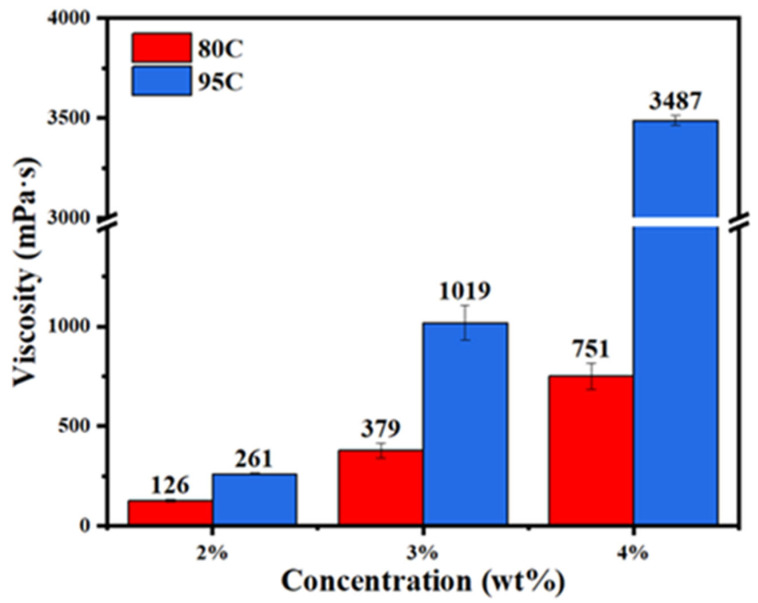
Viscosity of chitosan solutions with different degrees of deacetylation and concentrations.

The viscosity of chitosan solutions with varying degrees of deacetylation and concentrations was measured using an MCR302 rheometer, as shown in Figure 7. The results revealed that at the same concentration, chitosan with a higher degree of deacetylation exhibited higher viscosity than that with a lower degree of deacetylation. Similarly, for a fixed degree of deacetylation, the viscosity increased with increasing concentration. For instance, the viscosity of a 3% chitosan solution with a deacetylation degree > 95% reached 1019 mPa s, exceeding the 751 mPa s measured for a 4% chitosan solution with a deacetylation degree of 80–95%. These findings indicate that both higher deacetylation degree and increased concentration contribute to elevated solution viscosity, which in turn enhances pore filling, hydrogen bond formation, and film-forming ability on fiber surfaces. This trend in viscosity is consistent with the contact angle measurements discussed earlier.

### 3.6. Fourier Transform Infrared Spectroscopy (ATR-FTIR)

**Figure 8:**
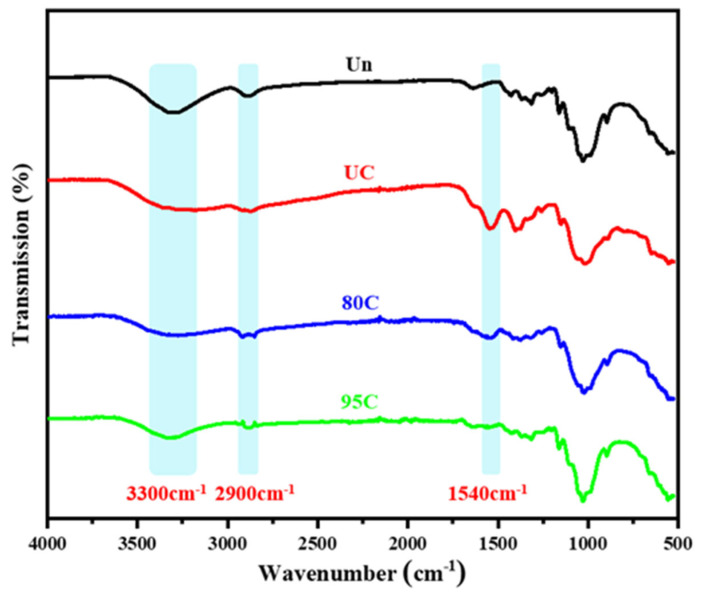
Infrared spectra of the uncoated meal box (Un), chitosan film (UC), and chitosan-coated meal boxes with different degrees of deacetylation (80C and 95C).

As shown in Figure 8, the Un, UC, 80C, and 95C samples exhibit a broad peak around 3300 cm^−1^, corresponding to the –OH stretching vibrations in cellulose and chitosan, as well as the –NH stretching vibrations in chitosan. The uncoated sample (Un) shows a larger peak area at 3300 cm^−1^ compared to the pure chitosan film (UC), indicating that cellulose contains more hydroxyl groups than chitosan’s hydroxyl and amino groups combined. The peak areas of the 80C and 95C samples in this region are smaller than that of the uncoated sample, suggesting that a portion of the surface hydroxyl groups was masked or reduced after coating with the chitosan solution. Since hydroxyl groups are typical hydrophilic groups, their reduction or coverage can enhance the water repellency of the coated meal boxes, which corresponds to the contact angle data to some extent.

The broad peak observed around 2900 cm^−1^ confirms the presence of –CH bonds. The absorption peaks at 1650 cm^−1^ and 1540 cm^−1^ correspond to the stretching vibrations of the amide I (C=O) and amide II (N–H/C–N) groups, respectively. These characteristic peaks closely match those of chitosan (CS). These findings confirm the successful coating of chitosan onto the surface of the meal boxes and partially explain the improvement in their water contact angle.

### 3.7. Scanning Electron Microscopy (SEM)

**Figure 9:**
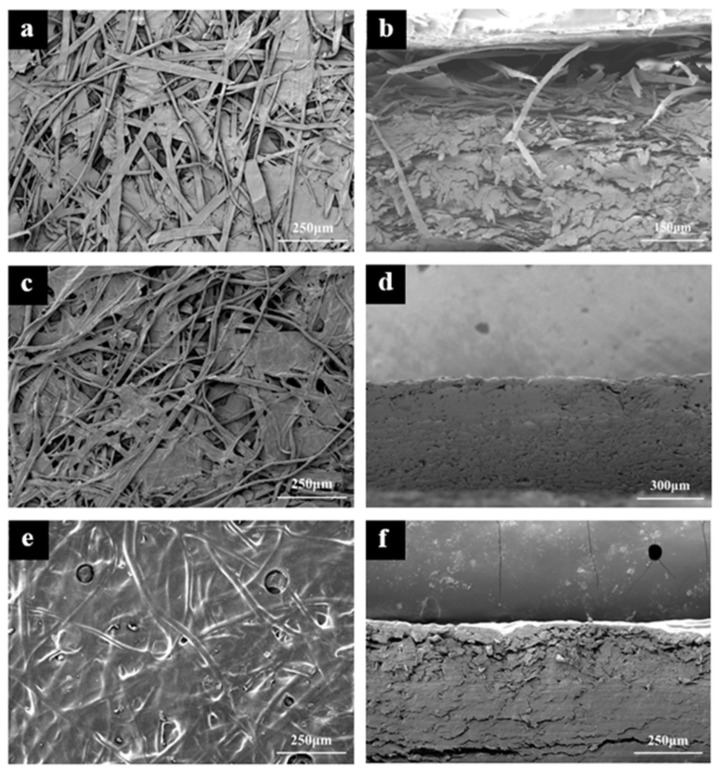
(**a**,**c**,**e**) Surface morphology of Un, 80C and 95C samples. (**b**,**d**,**f**) Cross-sectional morphology of Un, 80C and 95C samples.

The surface morphology of uncoated meal box samples (Un) and samples coated with chitosan of varying degrees of deacetylation (80C and 95C) was characterized using scanning electron microscopy (SEM), as presented in Figure 9. Figure 9a,b illustrates the surface and cross-sectional views of the uncoated sample, respectively. The microstructure reveals an interwoven network of cellulose microfibers with a loosely packed and highly porous architecture. This structure accounts for the poor water and oil repellency observed in uncoated meal boxes, as the abundant pores provide pathways for liquid infiltration.

Upon coating with chitosan of 80–95% deacetylation, the surface morphology (Figure 9c) shows partial filling of the surface pores, and a visible film of chitosan is observed on the fiber network. Correspondingly, the cross-sectional image in Figure 9d displays a significant compaction compared to the uncoated counterpart, suggesting that chitosan permeates and reinforces not only the surface but also the internal fiber network. This improved structural integrity contributes to enhanced water and oil barrier properties.

Figure 9e,f exhibits the surface and cross-section of the meal box coated with >95% deacetylated chitosan. The fiber matrix is uniformly covered, with near-complete pore filling and a continuous film observed across the surface. The cross-section shows a highly compact, dense structure. The superior film-forming and pore-filling capacity of highly deacetylated chitosan is attributed to its higher viscosity and stronger intermolecular hydrogen bonding, leading to significantly improved water and oil resistance compared to the lower deacetylation coating.

### 3.8. Mechanical Properties Analysis

**Figure 10:**
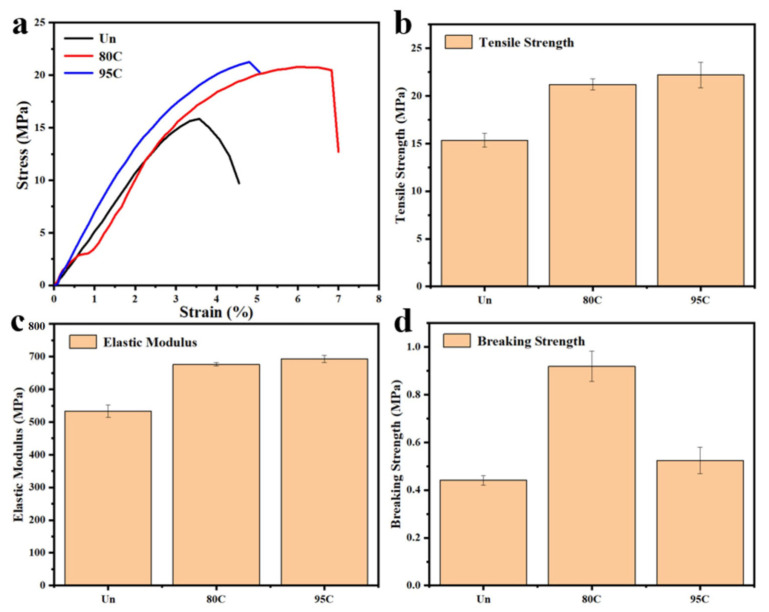
Mechanical properties of uncoated and chitosan-coated meal boxes with different degrees of deacetylation: (**a**) Stress–strain diagrams; (**b**) Tensile strength; (**c**) Elastic modulus; (**d**) Breaking strength.

The mechanical properties of uncoated samples and chitosan-coated counterparts with varying deacetylation degrees (80–95% and >95%) were evaluated using a universal material testing machine. As illustrated in Figure 10a–d, the analysis included stress–strain curves, tensile strength, elastic modulus, and fracture strength. The results consistently demonstrated enhanced mechanical performance in coated samples compared to uncoated ones. Specifically, the tensile strength increased from 15.85 MPa for uncoated samples to over 20 MPa for all coated variants. Furthermore, a gradual improvement in properties correlated with higher deacetylation degrees: samples with >95% deacetylation achieved a tensile strength of 21.26 MPa, slightly surpassing the 20.79 MPa observed in the 80–95% group.

### 3.9. Biodegradation Performance

**Figure 11:**
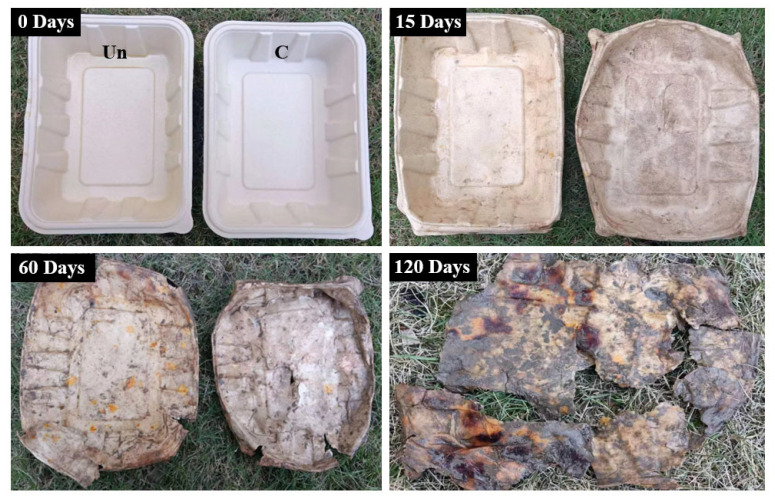
Biodegradation of uncoated and chitosan-coated meal boxes after 0, 15, 60, and 120 days.

The biodegradability of the uncoated and chitosan-coated samples was evaluated using a soil burial method. Samples were exhumed at specific intervals to assess the extent of degradation. As shown in Figure 11, photographs were taken at 0, 15, 60, and 120 days after burial. After 15 days, both types of samples began to deform. By 60 days, the sample edges had noticeably degraded and started to disintegrate. After 120 days, significant decomposition was observed, with samples breaking down into fragments. These results demonstrate that both the uncoated and chitosan-coated meal boxes exhibit excellent biodegradability and can decompose within a relatively short period.

### 3.10. Thermogravimetric (TGA) Analysis

**Figure 12:**
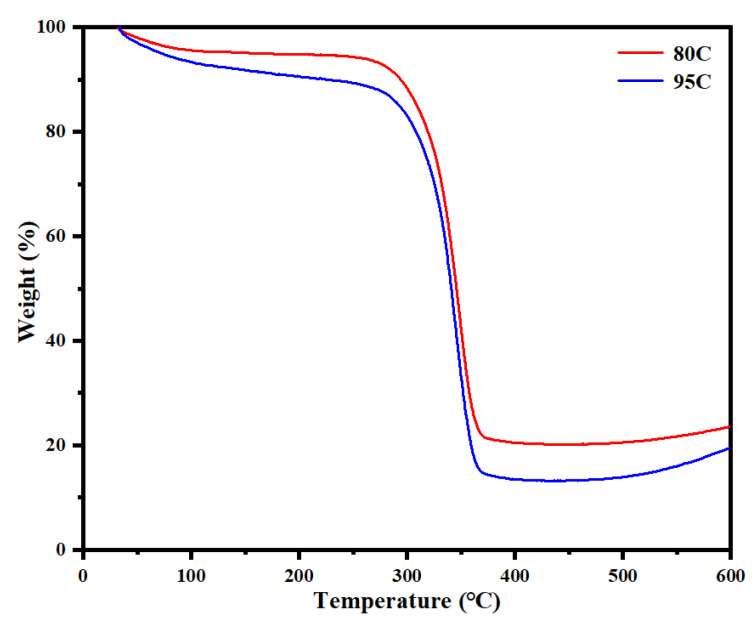
Thermogravimetric analysis spectra of chitosan-coated meal boxes with different degrees of deacetylation.

TGA was performed on chitosan-coated lunch boxes with different deacetylation degrees to evaluate their thermal stability. Figure 2, Figure 3, Figure 4, Figure 5, Figure 6, Figure 7, Figure 8, Figure 9, Figure 10, Figure 11 and Figure 12 illustrate the mass changes of 80–95% deacetylated chitosan-coated lunch box (80C) and >95% deacetylated chitosan-coated lunch box (95C) samples during heating from 30 °C to 600 °C. The mass loss below 130 °C can be attributed to moisture evaporation. All samples exhibited significant mass loss above 300 °C, primarily due to cellulose decomposition and the degradation of chitosan polymer units. Analysis of the TGA curves confirms that chitosan-coated lunch boxes remain highly stable below 300 °C, making them suitable for single-use food packaging applications.

### 3.11. Performance Indicator Analysis

**Figure 13:**
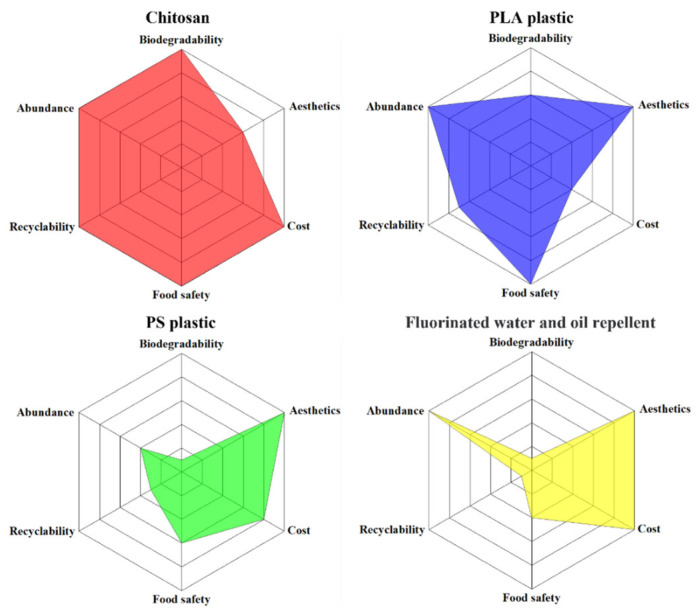
Comparison of the performance of chitosan-coated meal boxes with PLA plastic, PS plastic, and fluorinated water and oil repellents.

The chitosan-coated sample was evaluated and compared with PLA plastic, PS plastic, and fluorinated water- and oil-repellent agents in terms of biodegradability, aesthetics, cost, food safety, odor, and raw material availability, as illustrated in Figure 13 [27]. The chitosan-coated samples demonstrate notable advantages in biodegradability, cost-effectiveness, food safety, and raw material abundance. These characteristics suggest that chitosan coatings are a promising and sustainable alternative to conventional disposable plastic food packaging materials.

## 4. Conclusions

This study systematically investigated the water and oil repellency of pulp-molded meal boxes coated with chitosan solutions of varying deacetylation degrees and concentrations. The results demonstrate that chitosan coating significantly enhances the barrier properties of the meal boxes, with higher deacetylation degrees and concentrations yielding superior performance. Notably, the sample coated with 4% chitosan solution with a deacetylation degree >95% exhibited the best repellency. ATR-FTIR and SEM analyses confirmed the successful deposition of chitosan, which filled surface pores and formed a uniform film, thereby improving hydrophobicity. The increasing viscosity of the chitosan solution with higher deacetylation degrees and concentrations facilitated better coverage and film formation. Additionally, coated samples exhibited enhanced mechanical strength and excellent biodegradability in soil within 120 days, making them promising alternatives to disposable plastic packaging. Despite these achievements, challenges remain in improving the hot-water resistance and addressing color uniformity post-coating. Future work should focus on structural modifications and process optimization to further enhance performance. Overall, this study provides a theoretical foundation and practical guidance for developing green, biodegradable food packaging. Chitosan, as a natural and sustainable polymer, holds great potential to replace traditional plastics and contribute to environmental protection and sustainable development in the packaging industry.

## Figures and Tables

**Table 1:** Preparation methods for 12 different grades of solutions.

Level	Surface Tension [mN m^−1^]	Castor Oil [g]	Methylbenzene [mL]	N-Heptane [mL]
1	33.652	969.0	0	0
2	31.486	872.1	50	50
3	29.472	775.2	100	100
4	27.535	678.3	150	150
5	25.831	581.4	200	200
6	24.718	484.5	250	250
7	23.416	387.6	300	300
8	23.038	290.7	350	350
9	22.907	193.8	400	400
10	22.539	96.9	450	450
11	22.449	0	500	500
12	22.081	0	450	550

**Table 2:** Basis weight, coating load and material thickness of different samples.

Sample Name	Material Thickness [μm]	Coating Thickness [μm]	Basis Weight [g m^−2^]	Coating Load [g m^−2^]
Un	670	-	86.5 ± 3.3	-
80C-2	686	16	92.5 ± 4.2	6.0 ± 5.3
80C-3	683	13	92.1 ± 2.8	5.6 ± 4.1
80C-4	687	17	91.4 ± 2.5	4.9 ± 3.6
95C-2	685	15	91.9 ± 3.0	5.4 ± 3.8
95C-3	684	14	91.0 ± 1.4	4.4 ± 0.5
95C-4	682	12	90.2 ± 0.8	3.7 ± 0.3

Sample coding scheme based on chitosan deacetylation degree and solution concentration. Un denotes the uncoated sample, while 80C and 95C refer to chitosan with 80–95% and >95% degrees of deacetylation, respectively. The numbers 2, 3, and 4 represent the chitosan solution concentrations (*w*/*v*%) of 2%, 3%, and 4%, respectively. For example, 95C-4 indicates a sample coated with a 4% chitosan solution with >95% deacetylation.

## Data Availability

The original contributions presented in this study are included in the article. Further inquiries can be directed to the corresponding author.
